# PD47 - Visible mould and dampness are associated with new onset wheezing in New Zealand children

**DOI:** 10.1186/2045-7022-4-S1-P47

**Published:** 2014-02-28

**Authors:** Caroline Shorter, Julian Crane, Kristin Wickens, Nevil Pierse, Thorsten Stanley, Phillipa Barnes, Janice Kang, Philippa Howden-Chapman

**Affiliations:** 1 University of Otago, Welllington, New Zealand

## Background

New Zealand has high rates of asthma and a poor standard of housing, with inadequate insulation and heating leading to cold damp and often mouldy homes. The aim of the present study was to explore whether there was an association between indoor fungi and new onset wheezing in New Zealand children.

## Methods

We undertook an incident case control study of 450 children. Cases were children aged 1 – 6 years recently diagnosed with wheezing and requiring treatment in the past 12 months. Each case was area, age and gender matched to two control children with no history of wheezing. The extent of visible mould was scored and mould odour and leaks were examined in the homes of the children by parents and researchers using standard methods.

## Results

Researcher observed mould levels were found to be significantly higher in the bedrooms of wheezing children than control children, with every unit increase in the mould score having an increased odds ratio of wheezing of 1.28 (95% CI 1.16 – 1.42). Parental reported mould was strongly correlated with (r =0.46), but lower than researcher observed mould score (P≤0.05). However, like researcher observed mould, parental observations of reported mould were significantly higher in wheezing children’s bedrooms than in control children’s bedrooms, with an increased odds ratio of 1.30 (95% CI 1.16 – 1.46) for every one unit increase in the extent of mould observed. Mould odour and leaks were also significantly associated with new onset wheezing (odour OR 1.88, 95% CI 1.20 – 2.94, leaks OR 1.71, 95% CI 1.14 – 2.58).

## Conclusions

This study indicates there is a strong association of visible mould and dampness markers in the child’s bedroom with new onset wheezing in New Zealand children. However, further independent microbial markers need to be analysed to confirm the association.

